# Supplementary figures and images for: Complex effects of kinase localization revealed by compartment-specific regulation of protein kinase A activity
Source: eLife. 2022 Feb 24;11:e66869. doi: 10.7554/eLife.66869 (PMC8871369; doi:10.7554/eLife.66869)

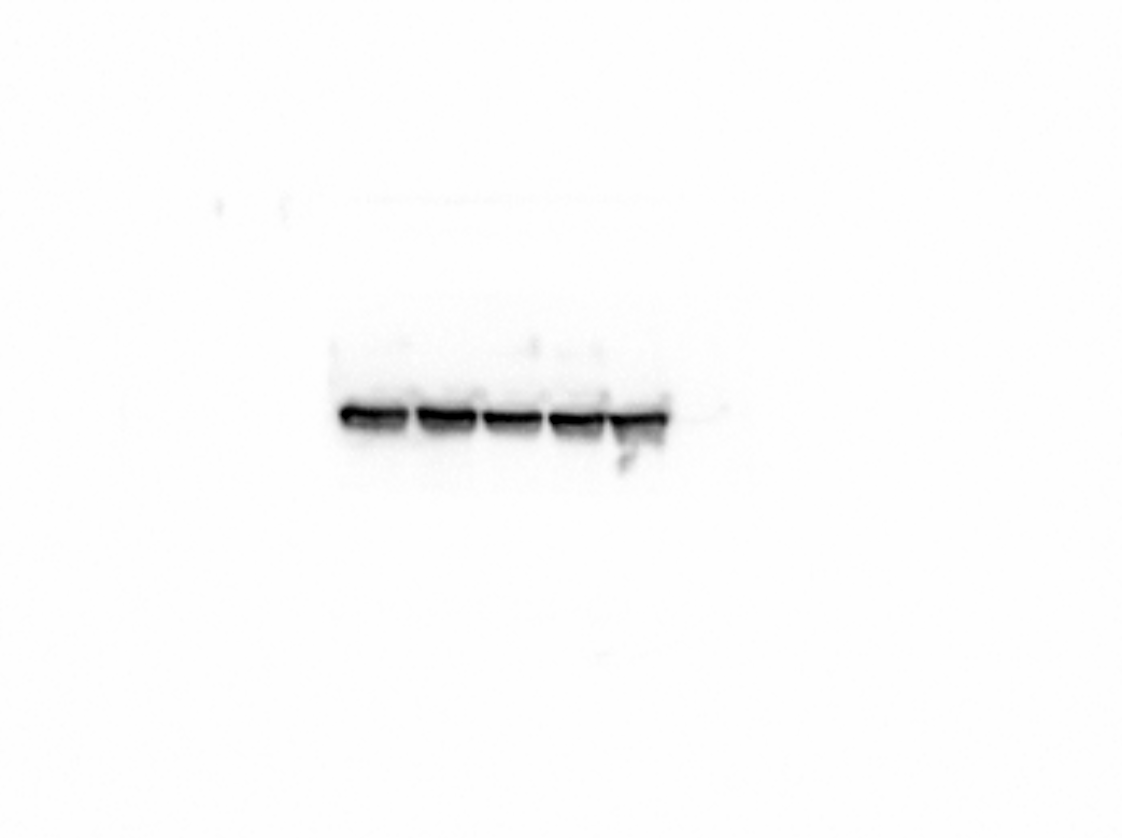

Supplement: Figure 1—figure supplement 1—source data 1. — Images are divided into two folders – one for each technical replicate. [file elife-66869-fig1-figsupp1-data1.zip › Set 1/Blot 1.1_GAPDH.tif]

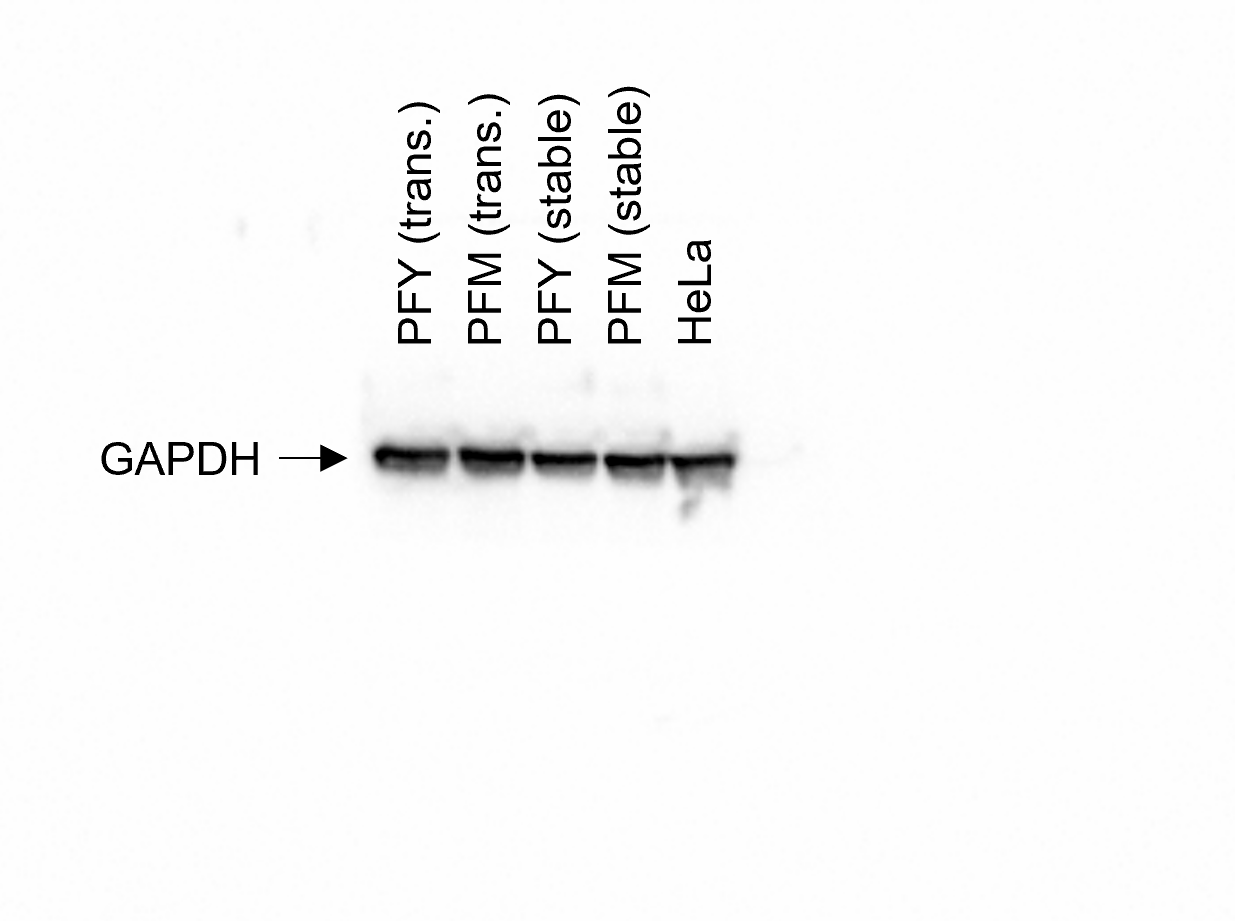

Supplement: Figure 1—figure supplement 1—source data 1. — Images are divided into two folders – one for each technical replicate. [file elife-66869-fig1-figsupp1-data1.zip › Set 1/Blot 1.1_GAPDH_labeled.tif]

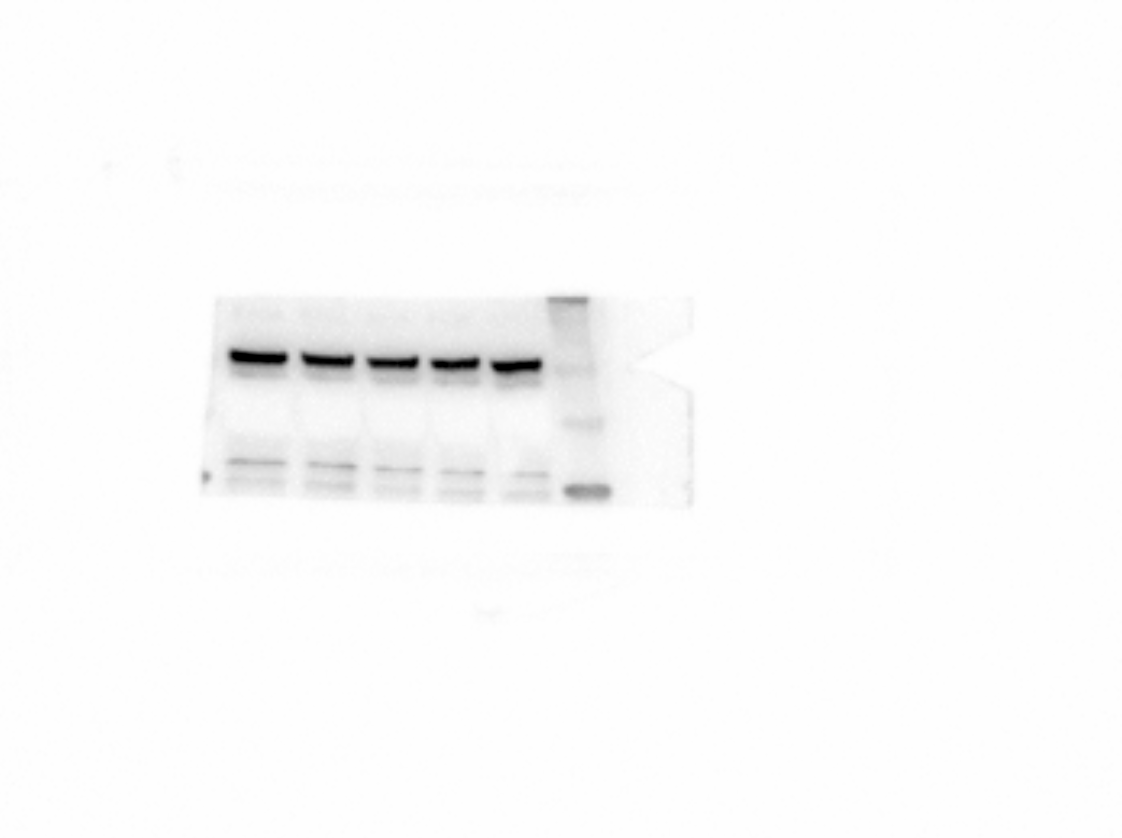

Supplement: Figure 1—figure supplement 1—source data 1. — Images are divided into two folders – one for each technical replicate. [file elife-66869-fig1-figsupp1-data1.zip › Set 1/Blot 1.1_PKARIIa.tif]

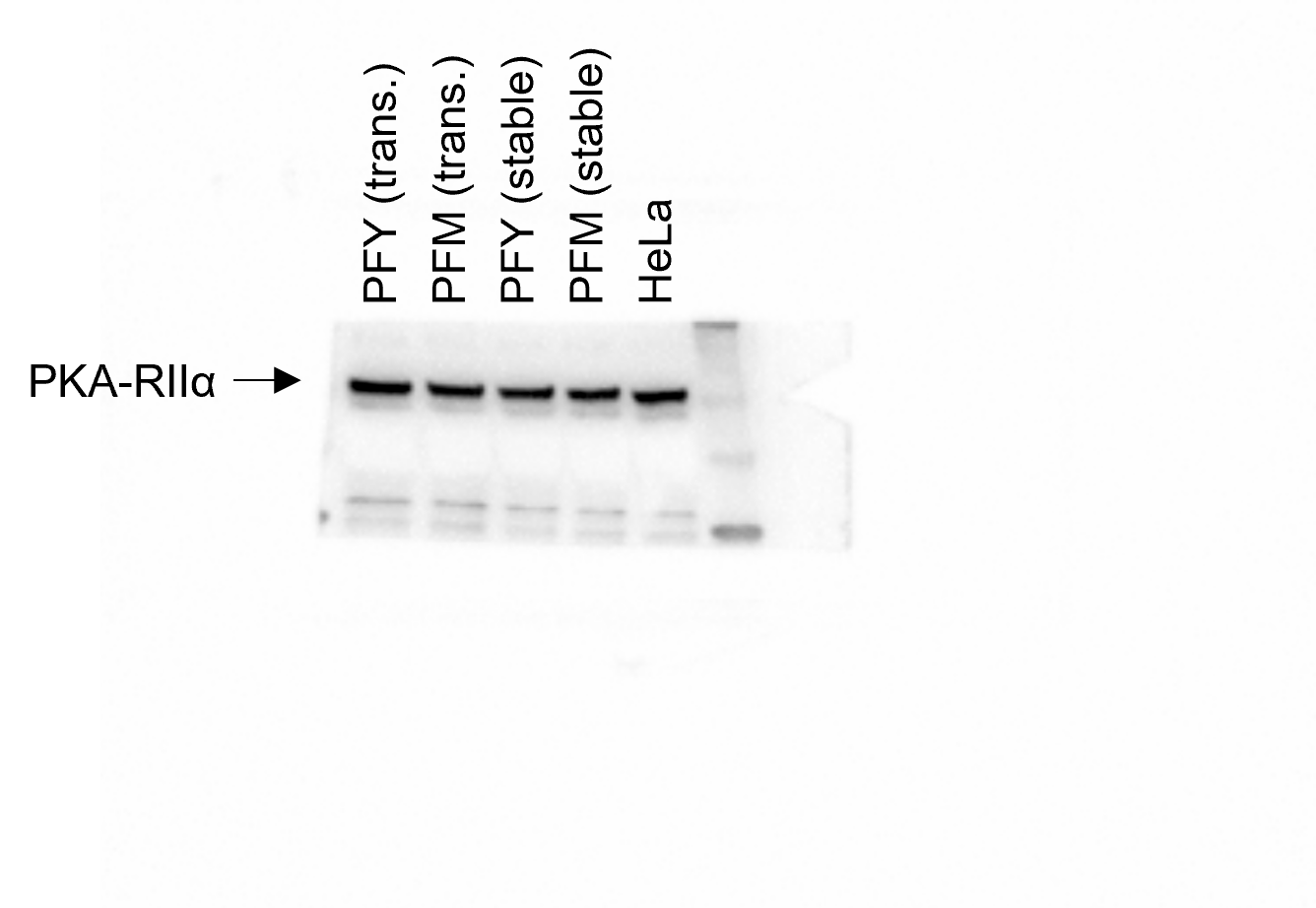

Supplement: Figure 1—figure supplement 1—source data 1. — Images are divided into two folders – one for each technical replicate. [file elife-66869-fig1-figsupp1-data1.zip › Set 1/Blot 1.1_PKARIIa_labeled.tif]

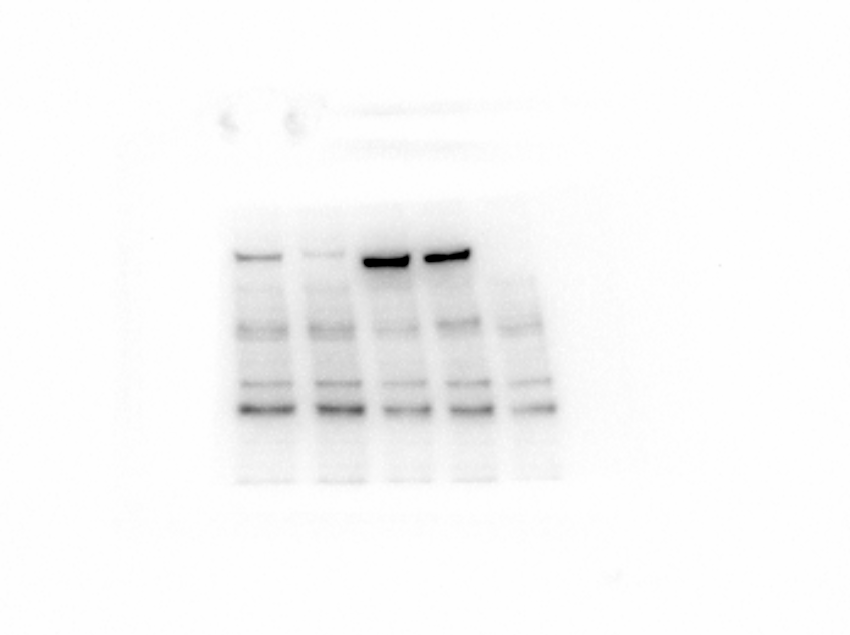

Supplement: Figure 1—figure supplement 1—source data 1. — Images are divided into two folders – one for each technical replicate. [file elife-66869-fig1-figsupp1-data1.zip › Set 1/Blot 1.1_PKARIIb.tif]

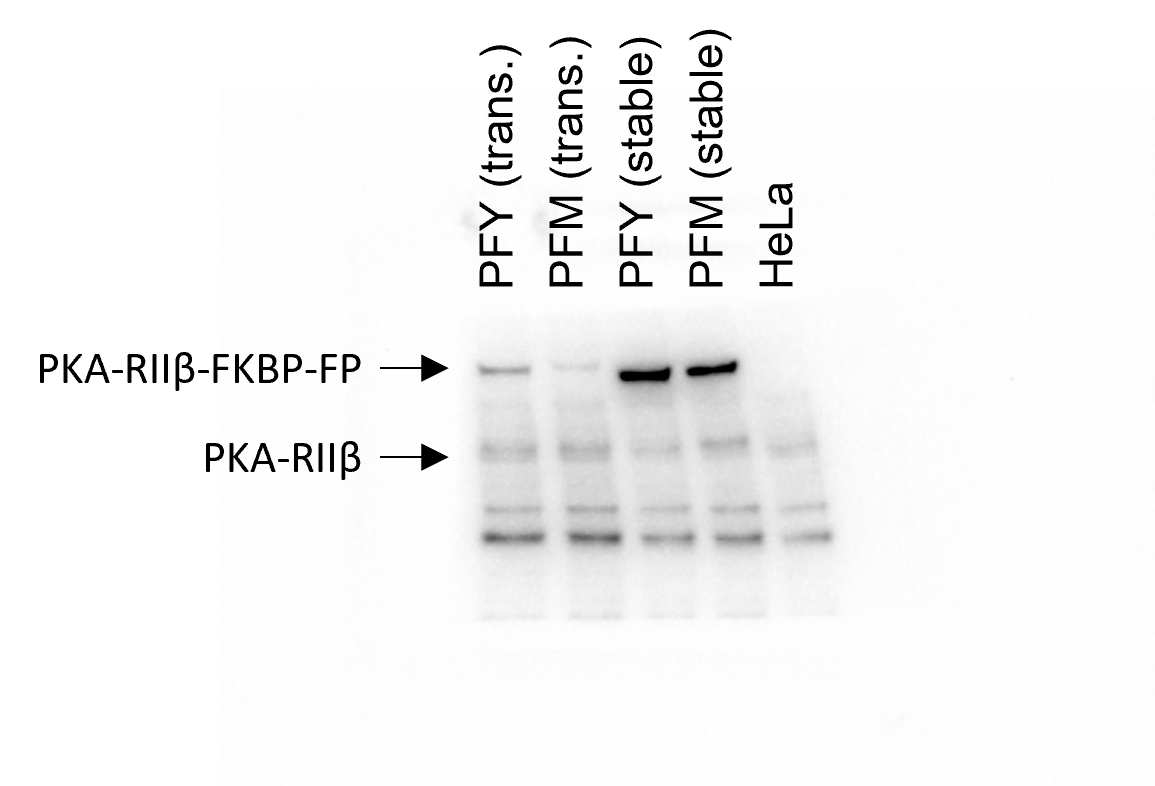

Supplement: Figure 1—figure supplement 1—source data 1. — Images are divided into two folders – one for each technical replicate. [file elife-66869-fig1-figsupp1-data1.zip › Set 1/Blot 1.1_PKARIIb_labeled.tif]

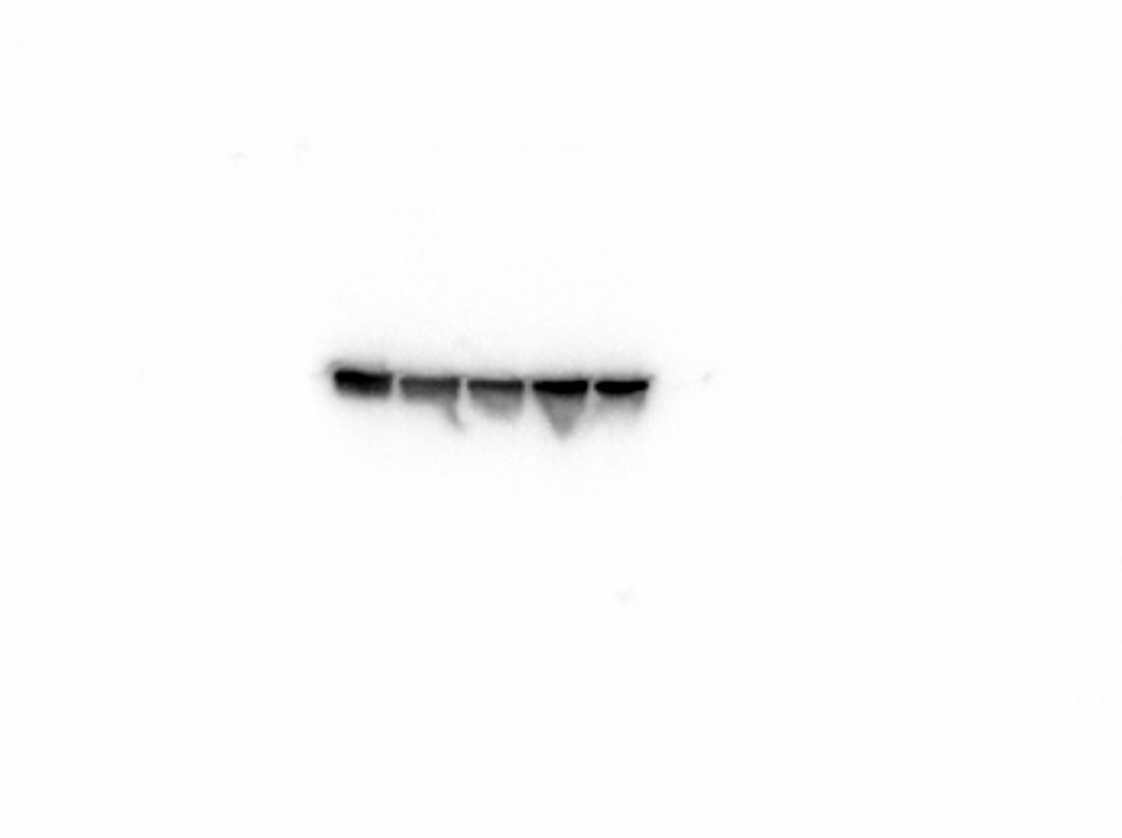

Supplement: Figure 1—figure supplement 1—source data 1. — Images are divided into two folders – one for each technical replicate. [file elife-66869-fig1-figsupp1-data1.zip › Set 1/Blot 1.2_GAPDH.tif]

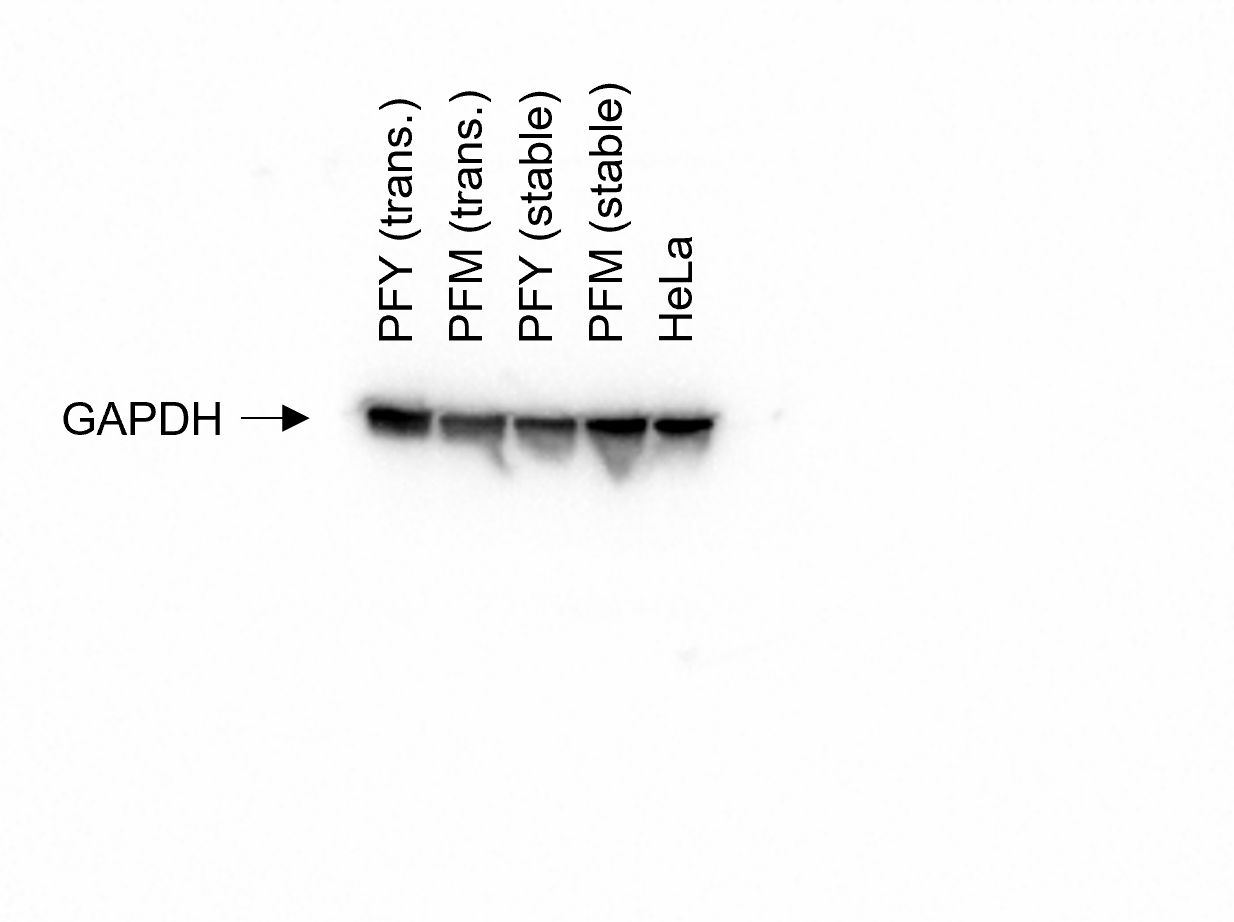

Supplement: Figure 1—figure supplement 1—source data 1. — Images are divided into two folders – one for each technical replicate. [file elife-66869-fig1-figsupp1-data1.zip › Set 1/Blot 1.2_GAPDH_labeled.tif]

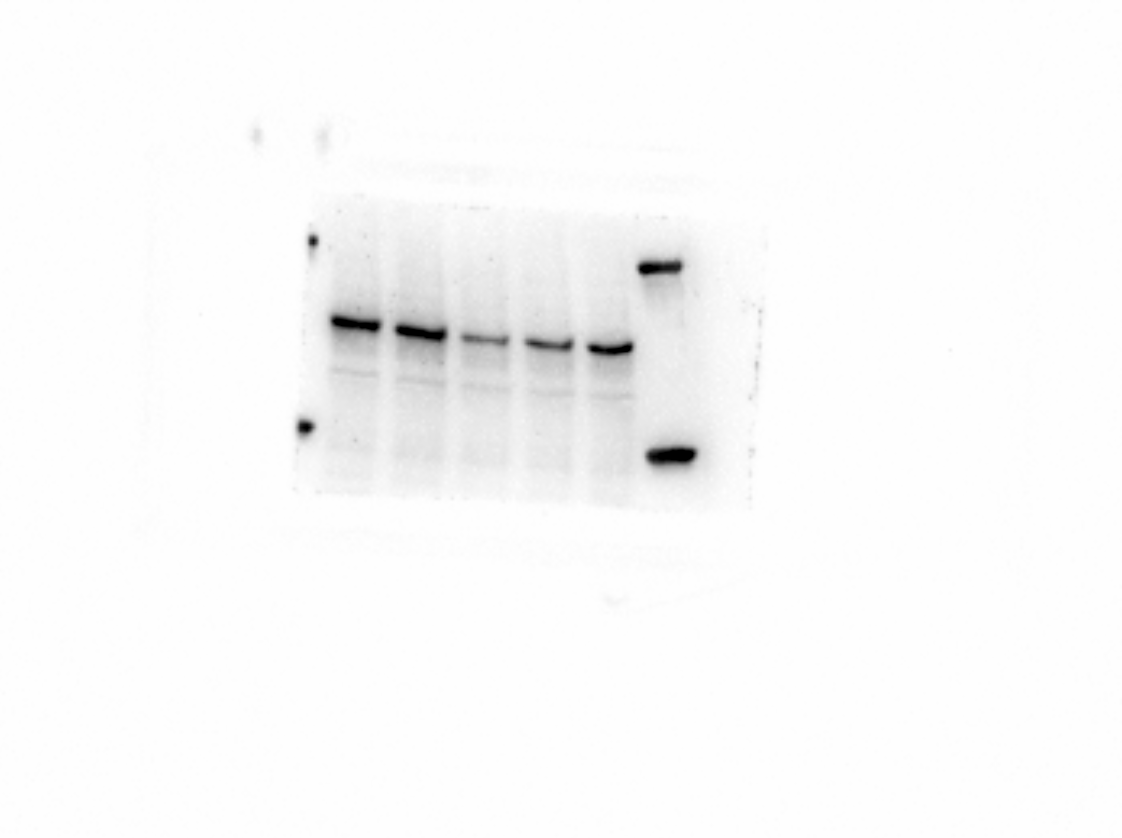

Supplement: Figure 1—figure supplement 1—source data 1. — Images are divided into two folders – one for each technical replicate. [file elife-66869-fig1-figsupp1-data1.zip › Set 1/Blot 1.2_PKARI.tif]

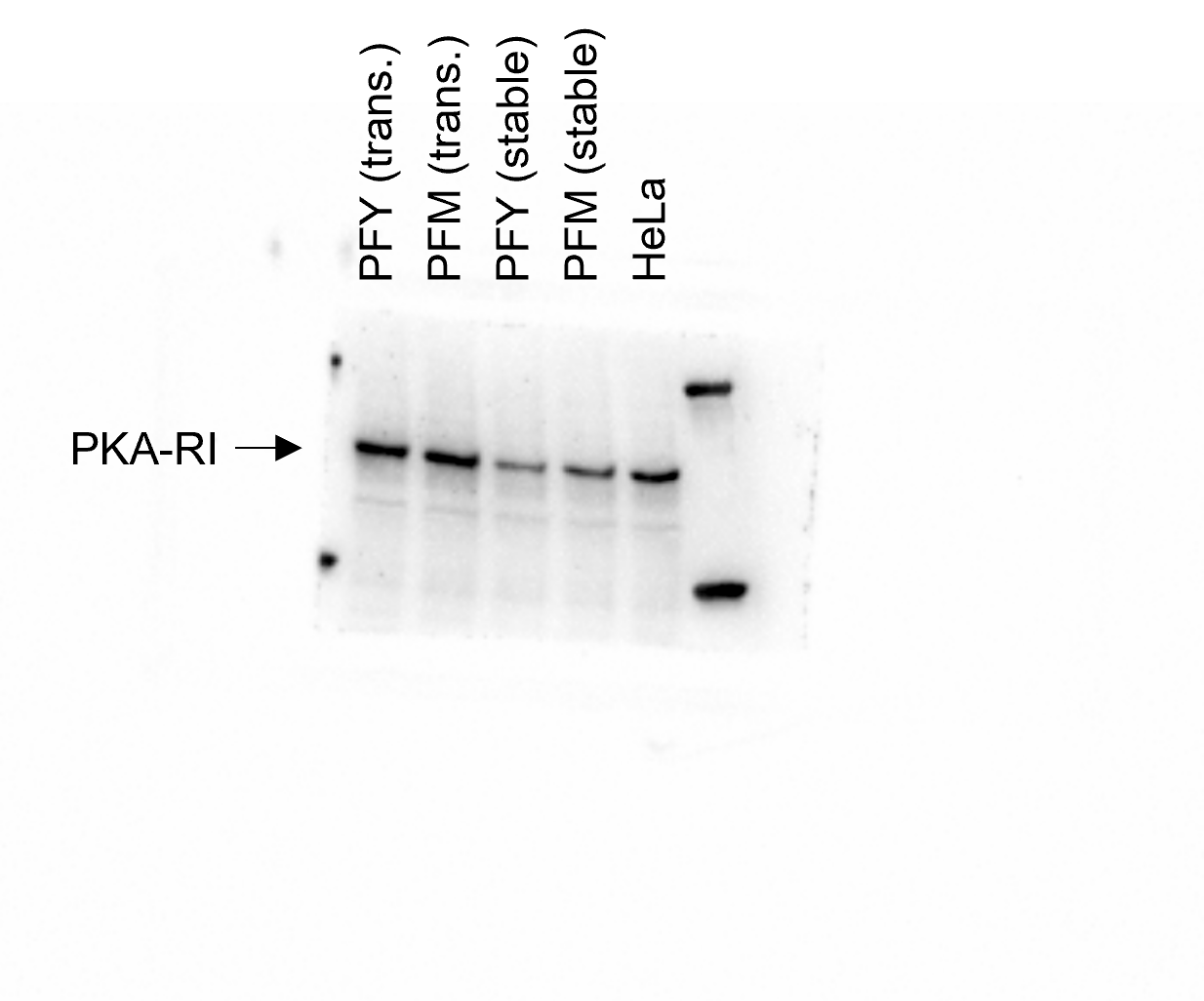

Supplement: Figure 1—figure supplement 1—source data 1. — Images are divided into two folders – one for each technical replicate. [file elife-66869-fig1-figsupp1-data1.zip › Set 1/Blot 1.2_PKARI_labeled.tif]

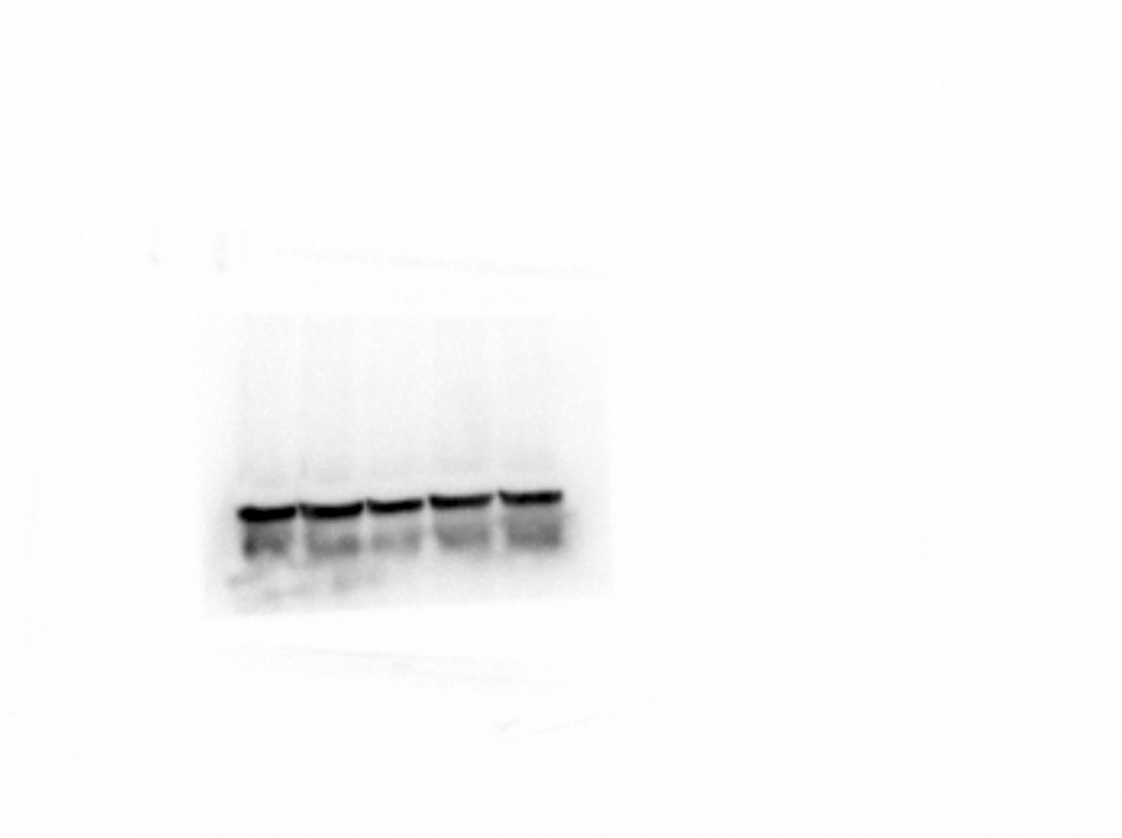

Supplement: Figure 1—figure supplement 1—source data 1. — Images are divided into two folders – one for each technical replicate. [file elife-66869-fig1-figsupp1-data1.zip › Set 1/Blot 1.3_GAPDH.tif]

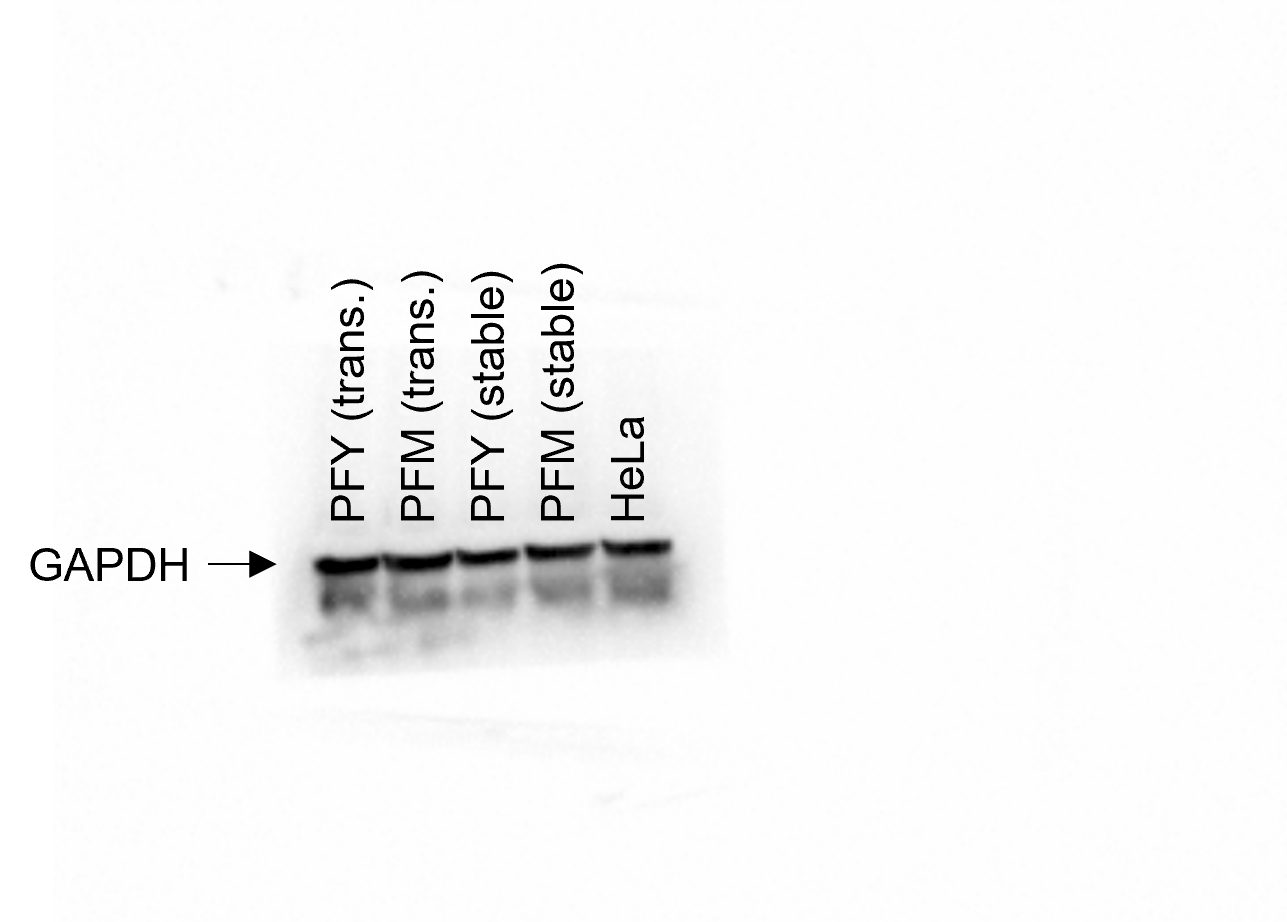

Supplement: Figure 1—figure supplement 1—source data 1. — Images are divided into two folders – one for each technical replicate. [file elife-66869-fig1-figsupp1-data1.zip › Set 1/Blot 1.3_GAPDH_labeled.tif]

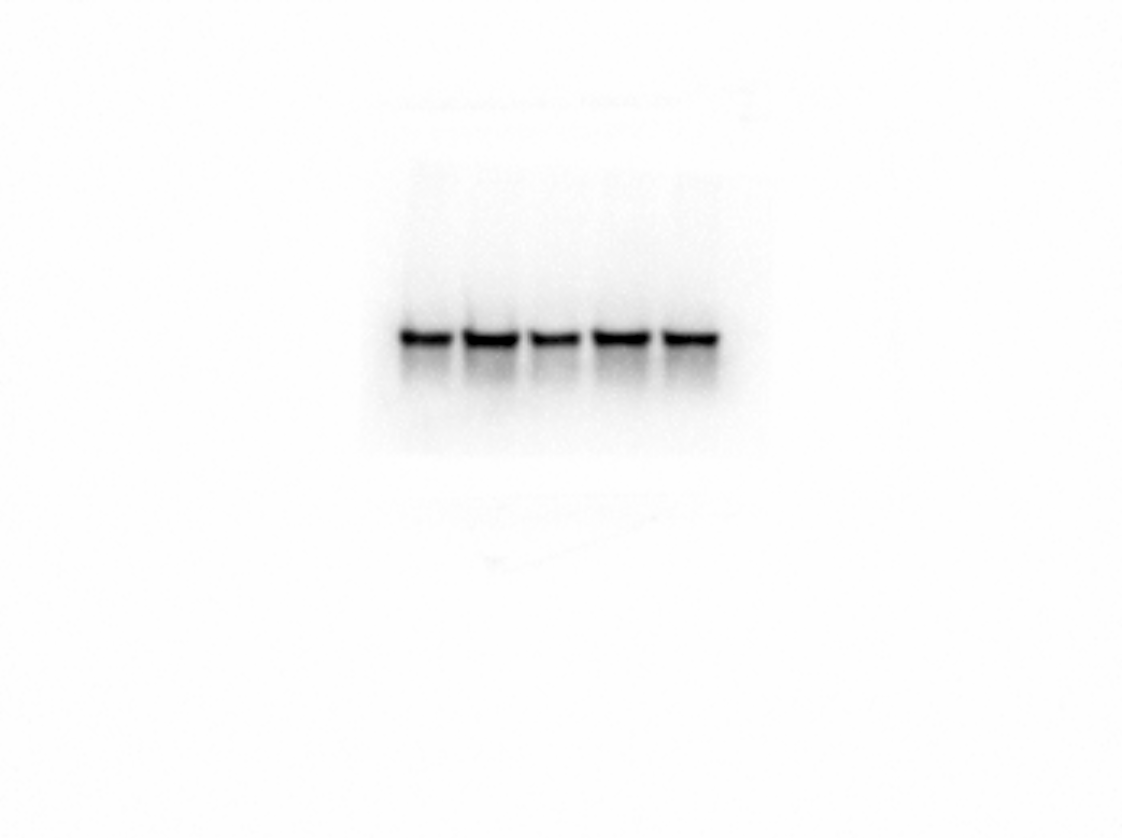

Supplement: Figure 1—figure supplement 1—source data 1. — Images are divided into two folders – one for each technical replicate. [file elife-66869-fig1-figsupp1-data1.zip › Set 1/Blot 1.3_PKAC.tif]

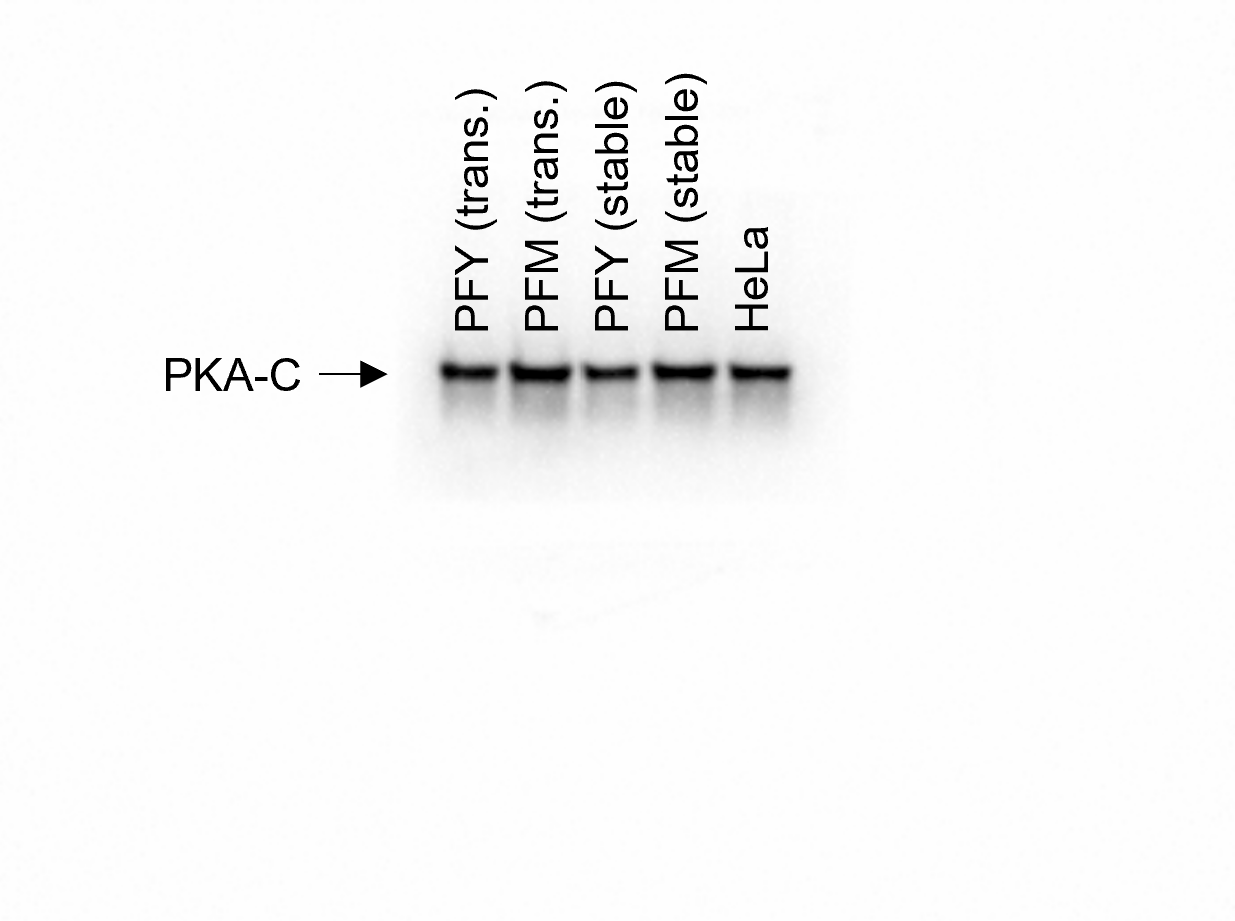

Supplement: Figure 1—figure supplement 1—source data 1. — Images are divided into two folders – one for each technical replicate. [file elife-66869-fig1-figsupp1-data1.zip › Set 1/Blot 1.3_PKAC_labeled.tif]

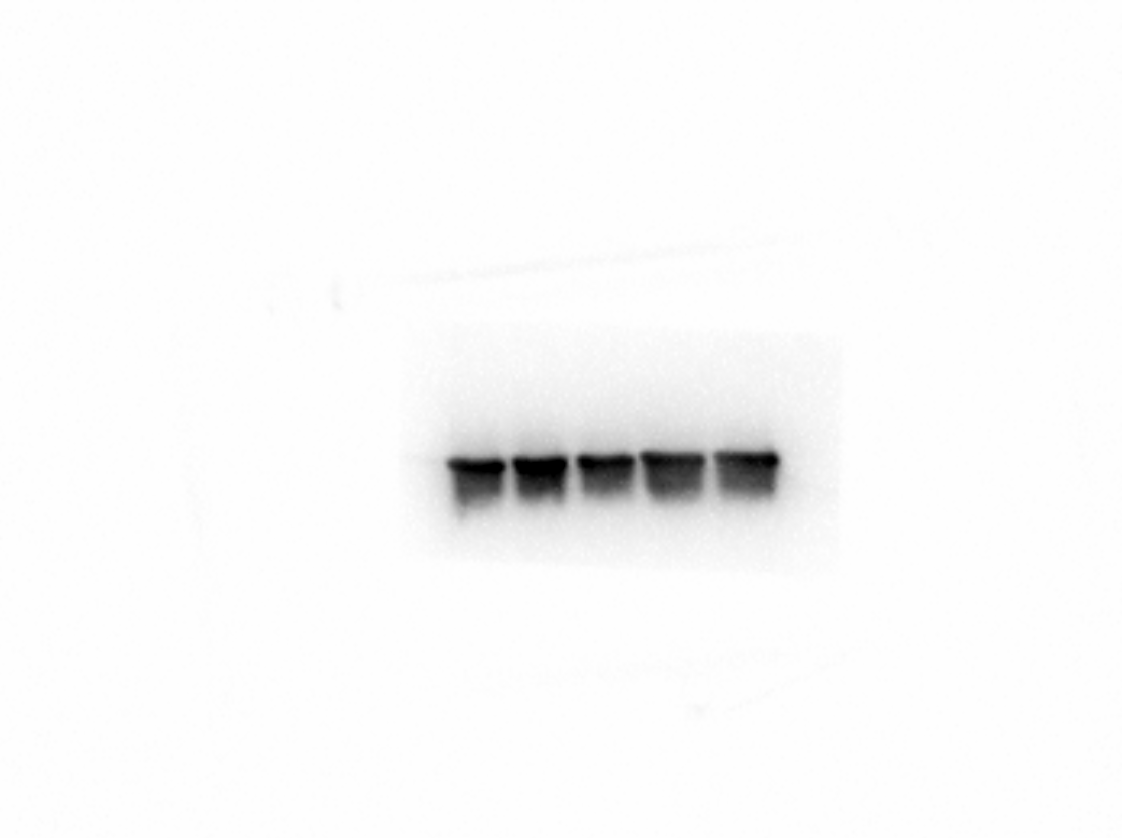

Supplement: Figure 1—figure supplement 1—source data 1. — Images are divided into two folders – one for each technical replicate. [file elife-66869-fig1-figsupp1-data1.zip › Set 2/Blot 2.1_GAPDH.tif]

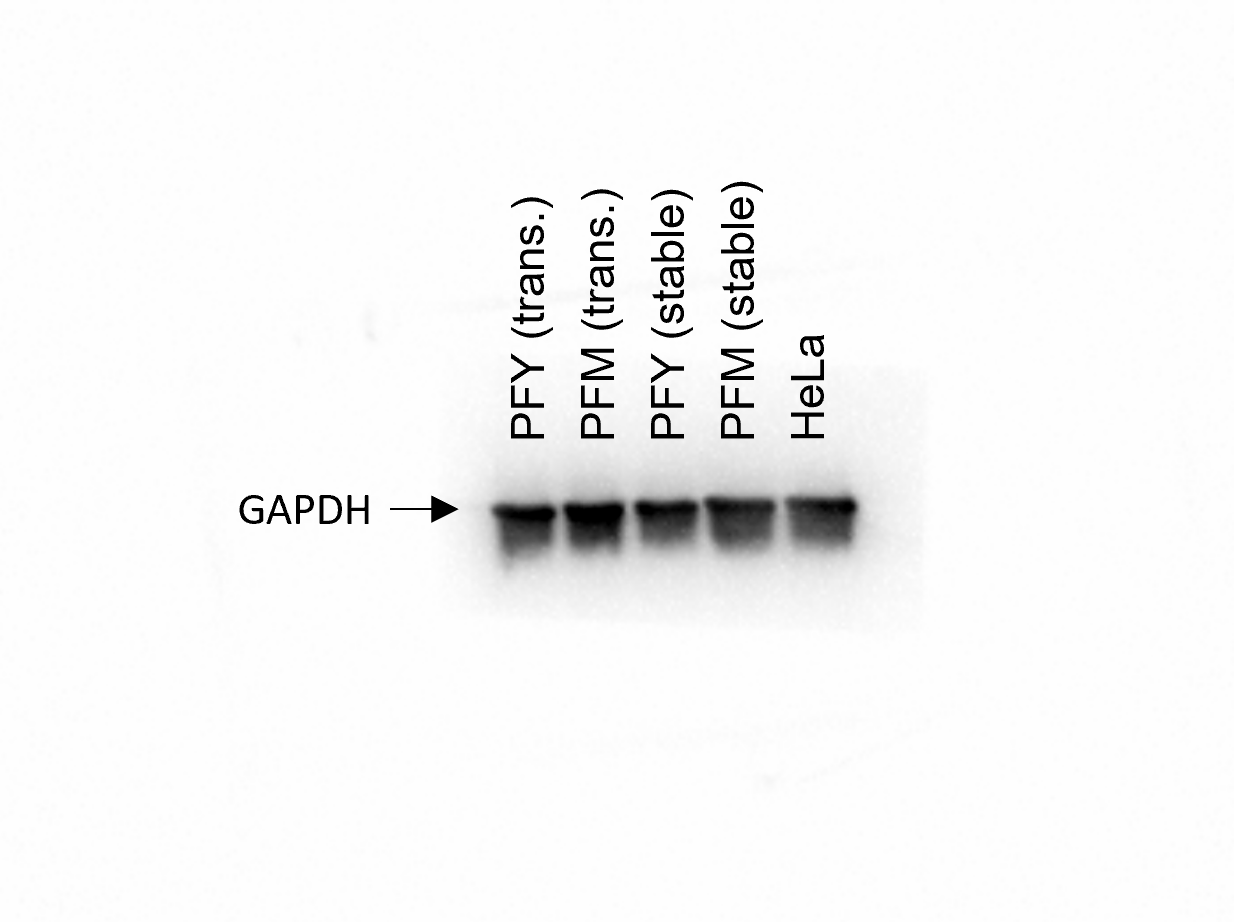

Supplement: Figure 1—figure supplement 1—source data 1. — Images are divided into two folders – one for each technical replicate. [file elife-66869-fig1-figsupp1-data1.zip › Set 2/Blot 2.1_GAPDH_labeled.tif]

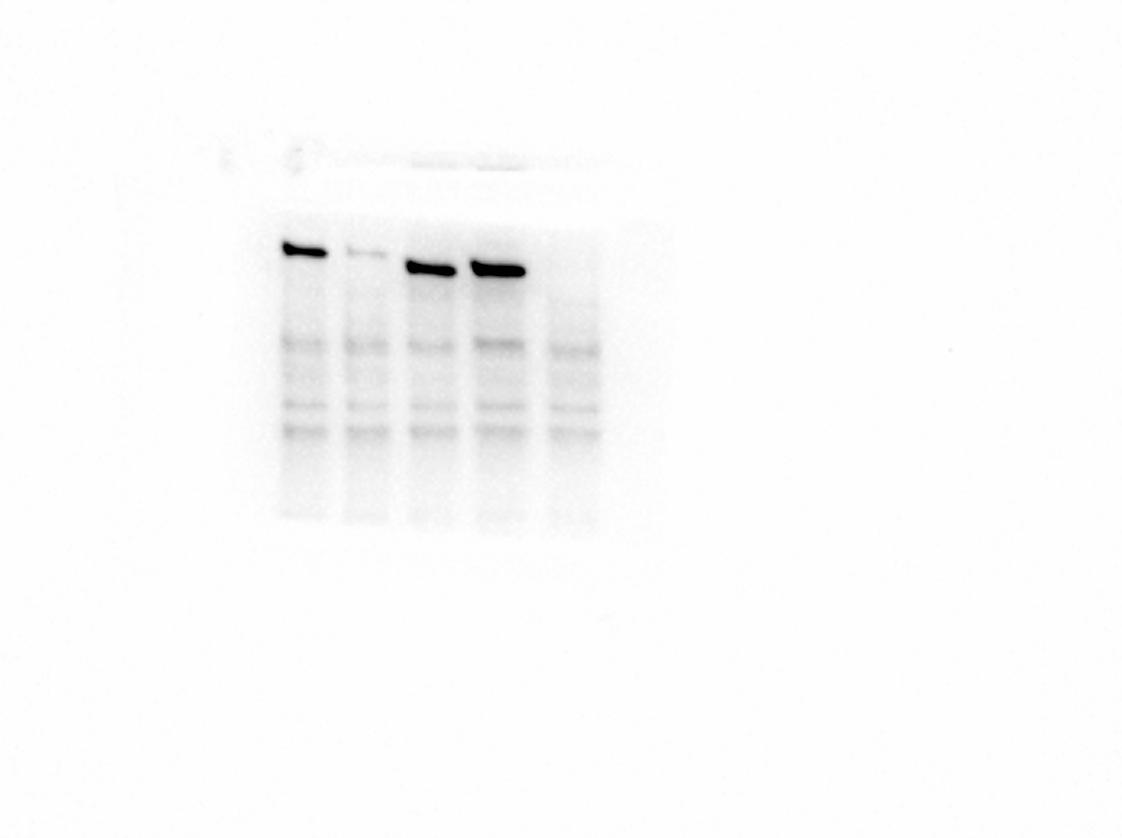

Supplement: Figure 1—figure supplement 1—source data 1. — Images are divided into two folders – one for each technical replicate. [file elife-66869-fig1-figsupp1-data1.zip › Set 2/Blot 2.1_PKARIIb.tif]

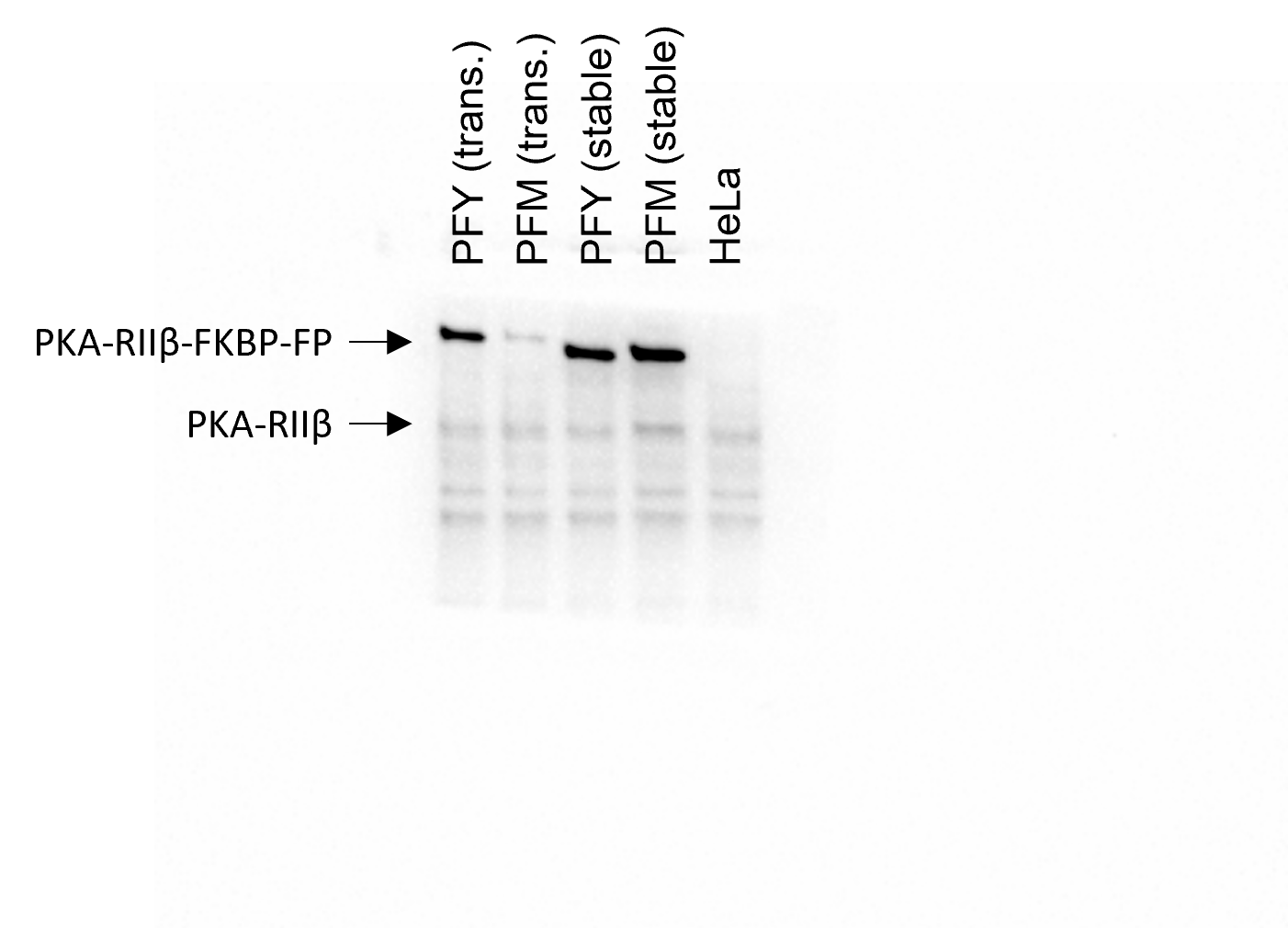

Supplement: Figure 1—figure supplement 1—source data 1. — Images are divided into two folders – one for each technical replicate. [file elife-66869-fig1-figsupp1-data1.zip › Set 2/Blot 2.1_PKARIIb_labeled.tif]

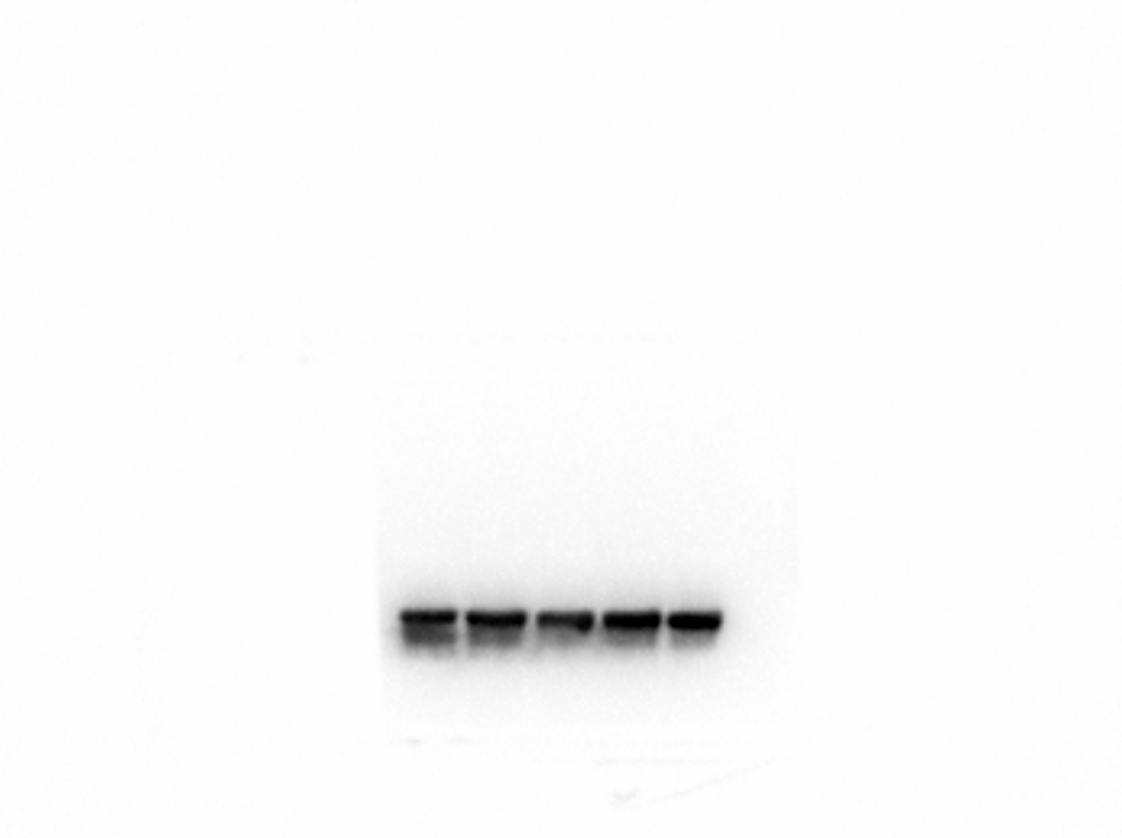

Supplement: Figure 1—figure supplement 1—source data 1. — Images are divided into two folders – one for each technical replicate. [file elife-66869-fig1-figsupp1-data1.zip › Set 2/Blot 2.2_GAPDH.tif]

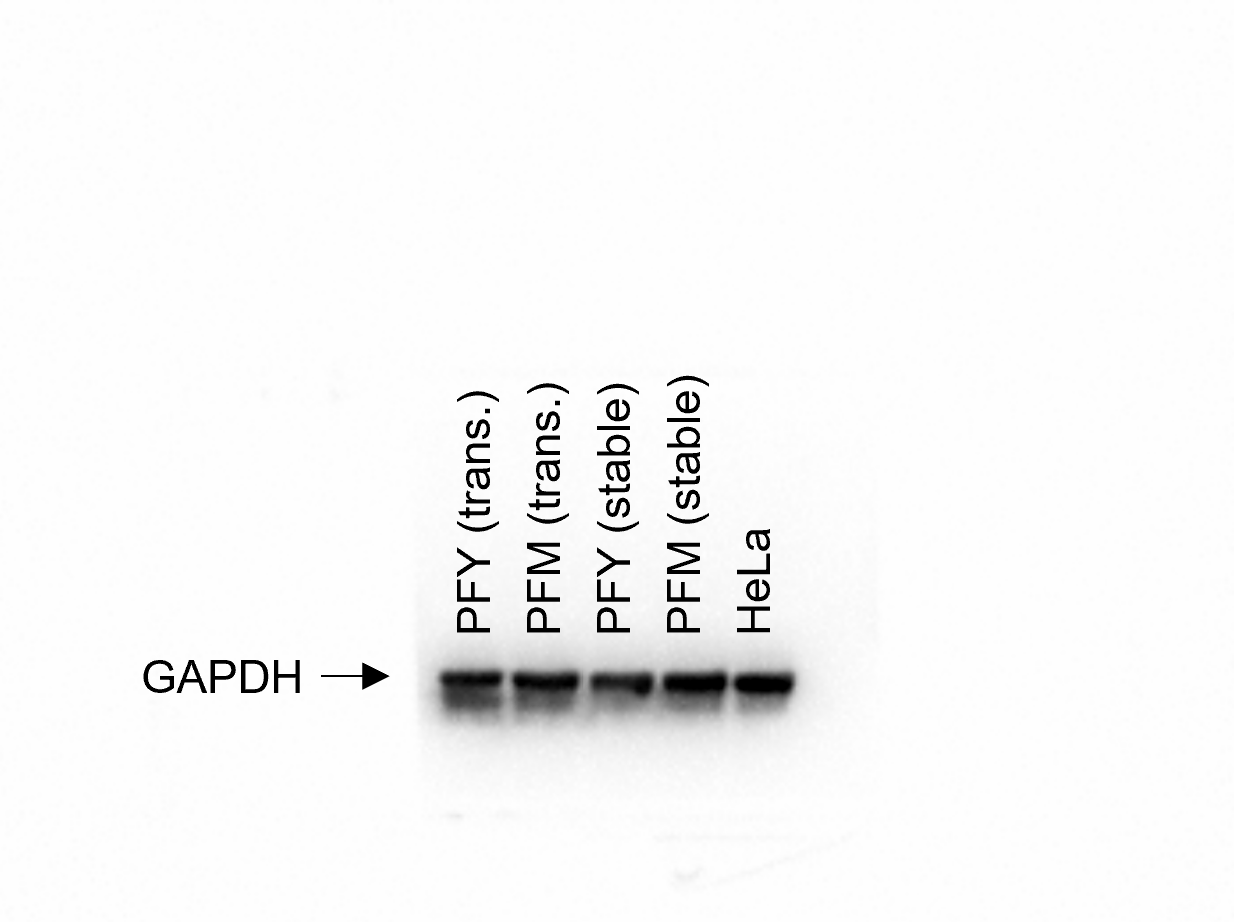

Supplement: Figure 1—figure supplement 1—source data 1. — Images are divided into two folders – one for each technical replicate. [file elife-66869-fig1-figsupp1-data1.zip › Set 2/Blot 2.2_GAPDH_labeled.tif]

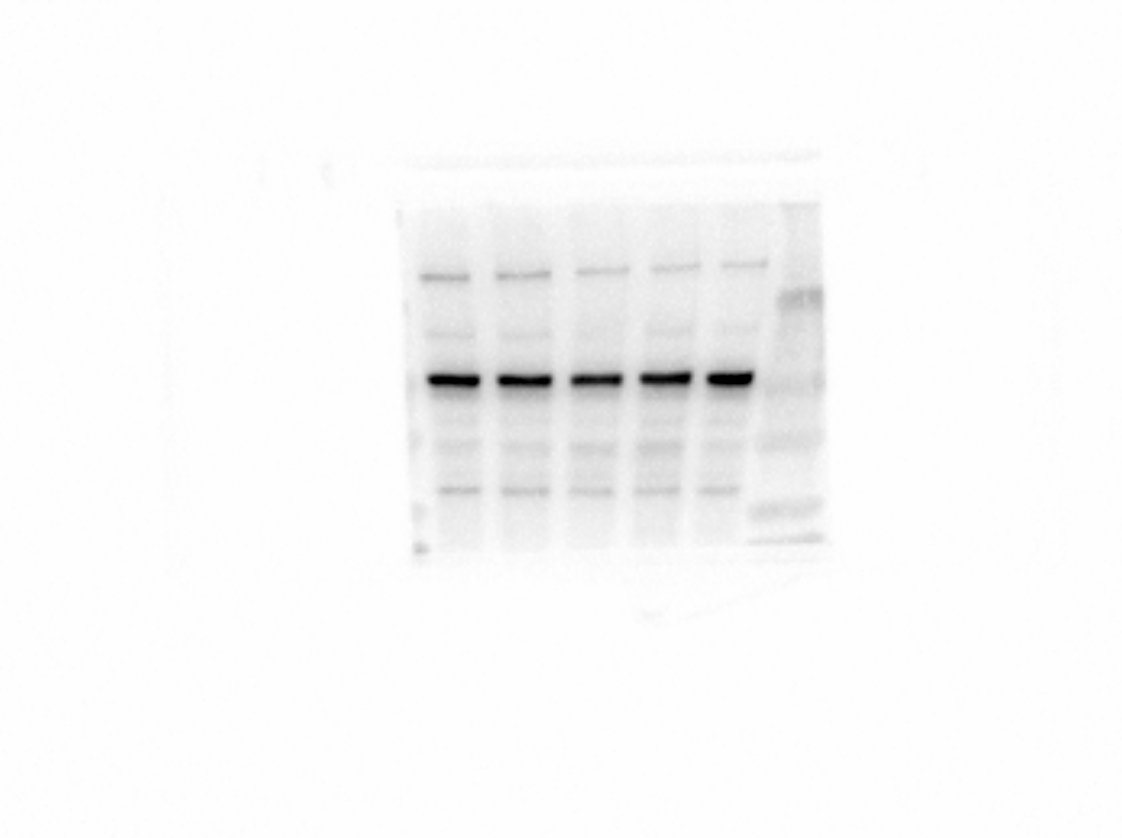

Supplement: Figure 1—figure supplement 1—source data 1. — Images are divided into two folders – one for each technical replicate. [file elife-66869-fig1-figsupp1-data1.zip › Set 2/Blot 2.2_PKARIIa.tif]

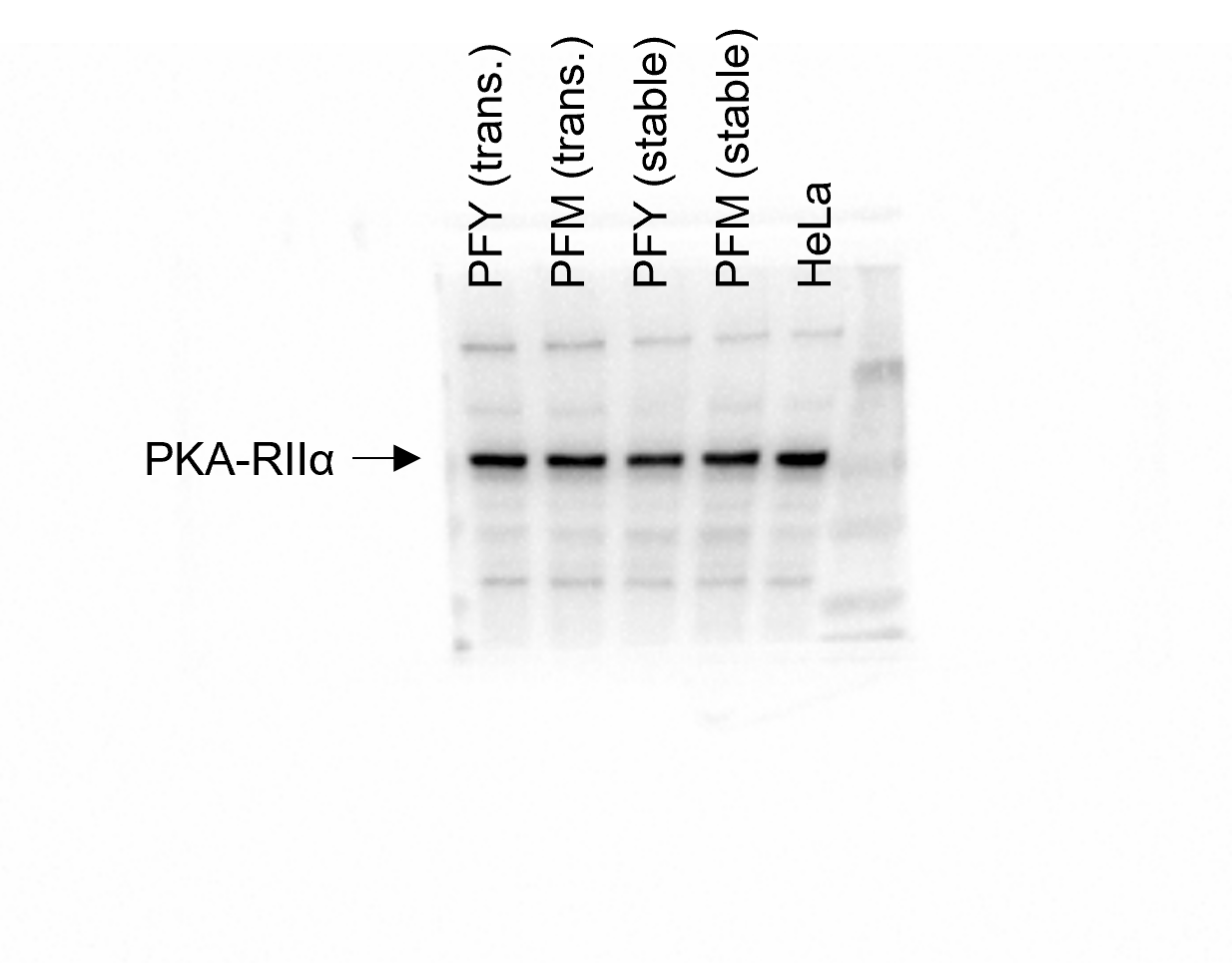

Supplement: Figure 1—figure supplement 1—source data 1. — Images are divided into two folders – one for each technical replicate. [file elife-66869-fig1-figsupp1-data1.zip › Set 2/Blot 2.2_PKARIIa_labeled.tif]

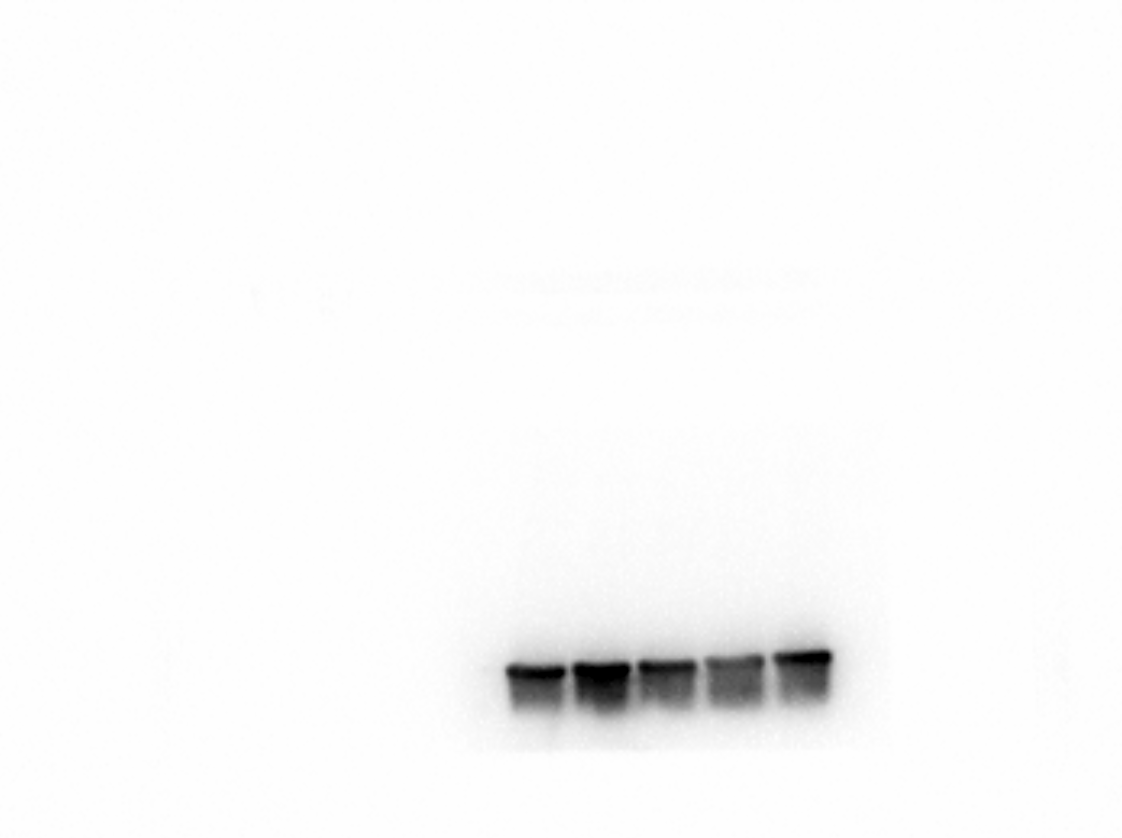

Supplement: Figure 1—figure supplement 1—source data 1. — Images are divided into two folders – one for each technical replicate. [file elife-66869-fig1-figsupp1-data1.zip › Set 2/Blot 2.3_GAPDH.tif]

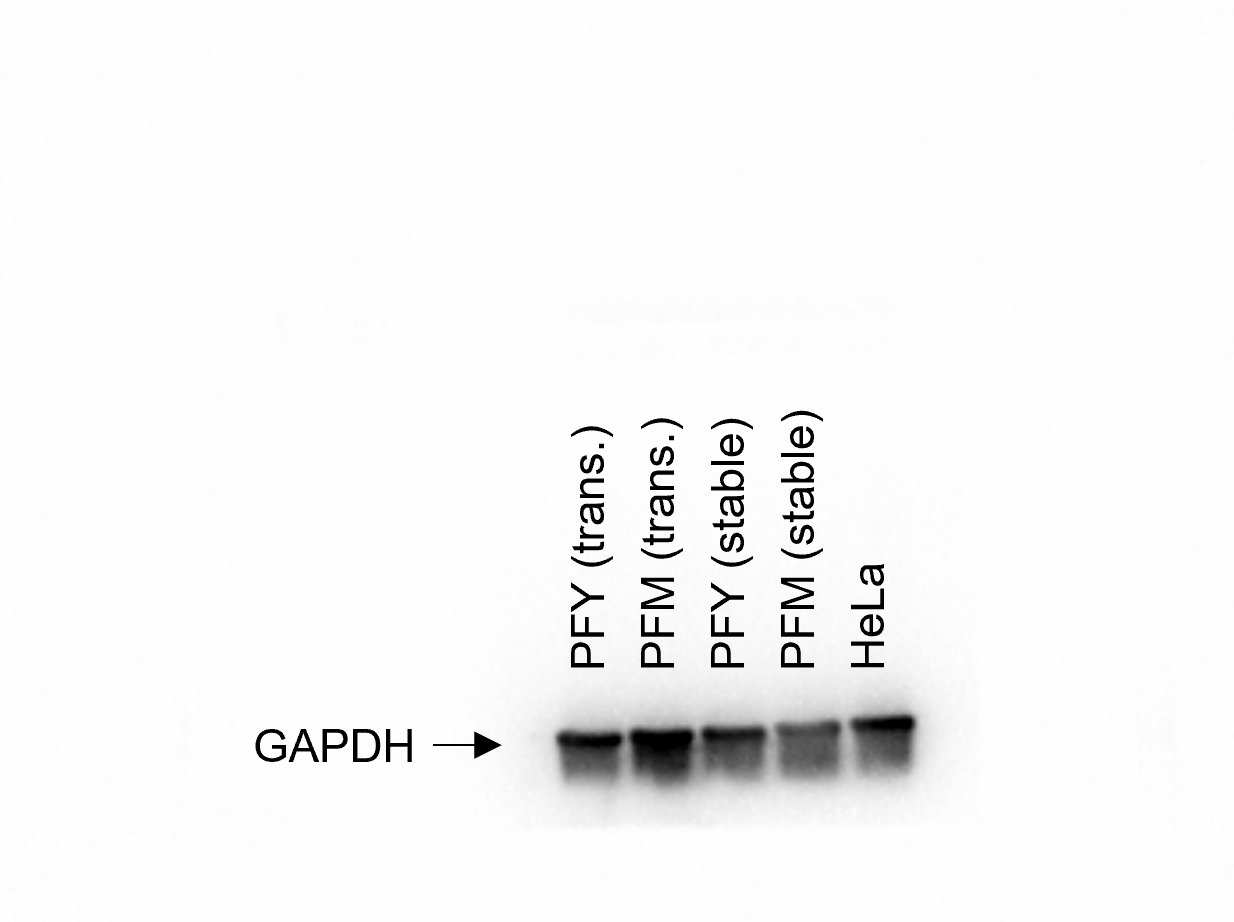

Supplement: Figure 1—figure supplement 1—source data 1. — Images are divided into two folders – one for each technical replicate. [file elife-66869-fig1-figsupp1-data1.zip › Set 2/Blot 2.3_GAPDH_labeled.tif]

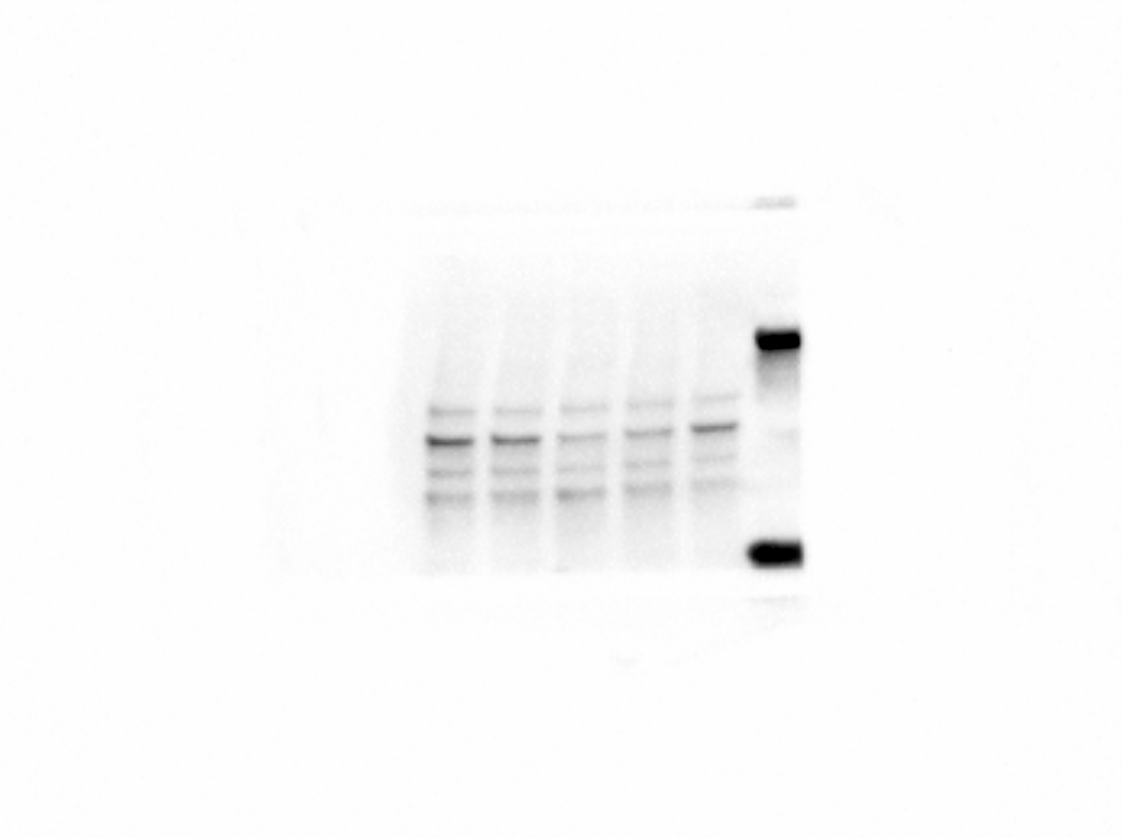

Supplement: Figure 1—figure supplement 1—source data 1. — Images are divided into two folders – one for each technical replicate. [file elife-66869-fig1-figsupp1-data1.zip › Set 2/Blot 2.3_PKARI.tif]

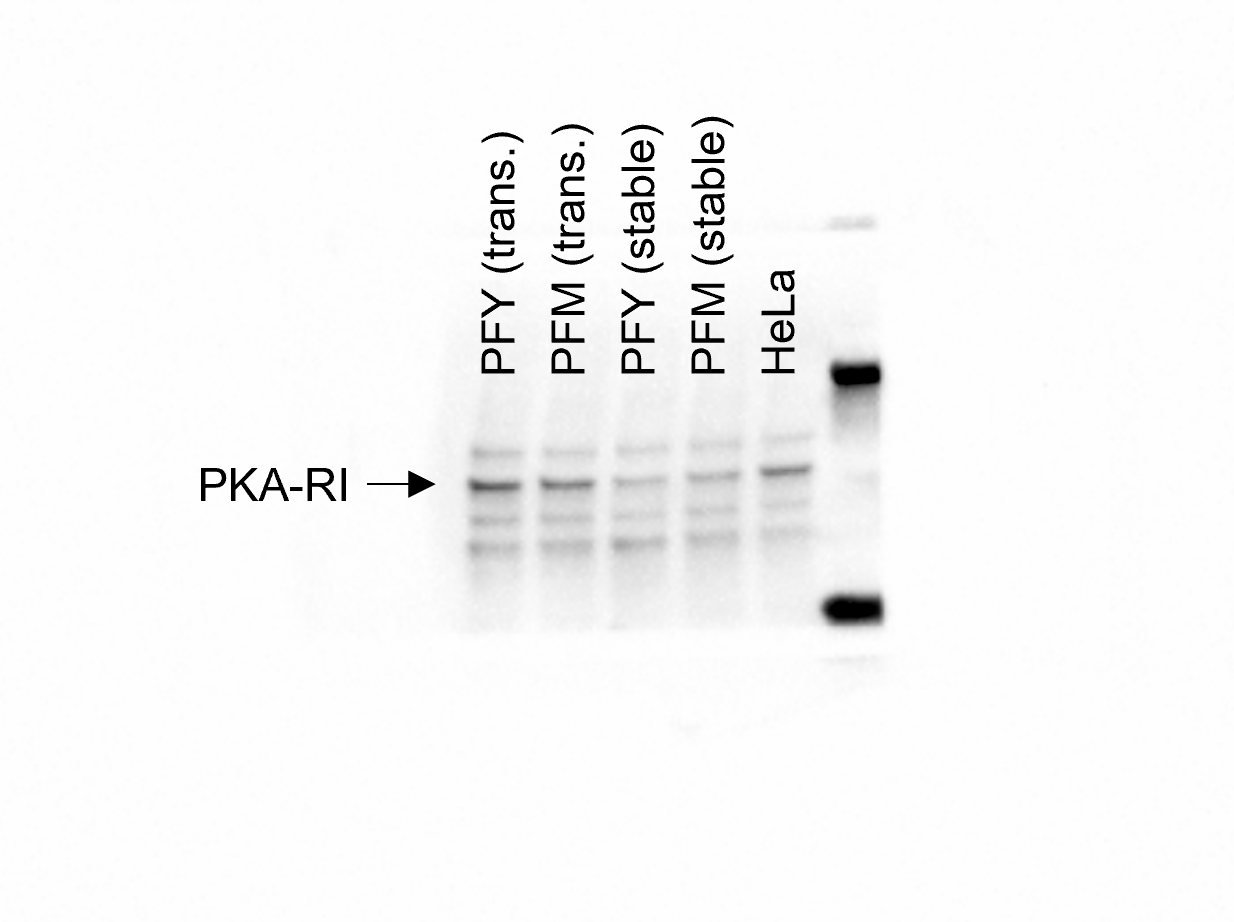

Supplement: Figure 1—figure supplement 1—source data 1. — Images are divided into two folders – one for each technical replicate. [file elife-66869-fig1-figsupp1-data1.zip › Set 2/Blot 2.3_PKARI_labeled.tif]

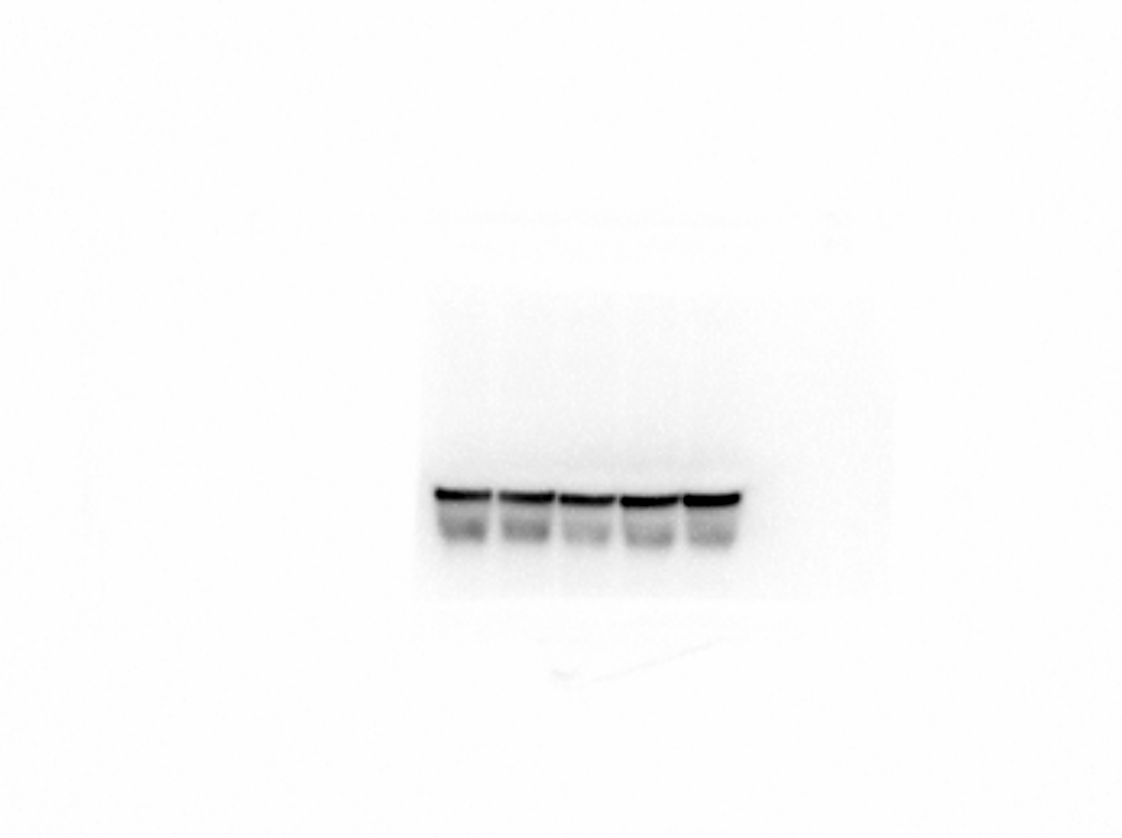

Supplement: Figure 1—figure supplement 1—source data 1. — Images are divided into two folders – one for each technical replicate. [file elife-66869-fig1-figsupp1-data1.zip › Set 2/Blot 2.4_GAPDH.tif]

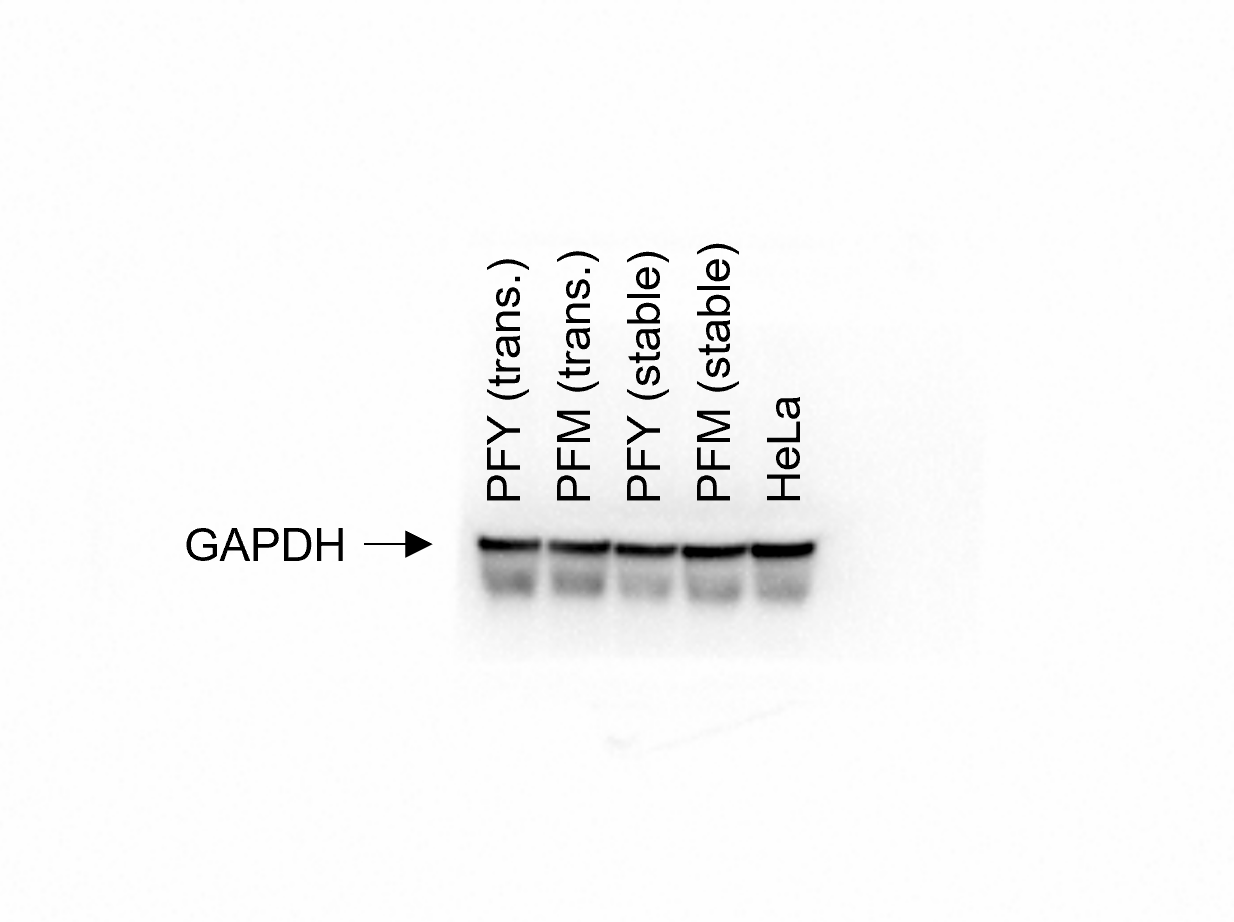

Supplement: Figure 1—figure supplement 1—source data 1. — Images are divided into two folders – one for each technical replicate. [file elife-66869-fig1-figsupp1-data1.zip › Set 2/Blot 2.4_GAPDH_labeled.tif]

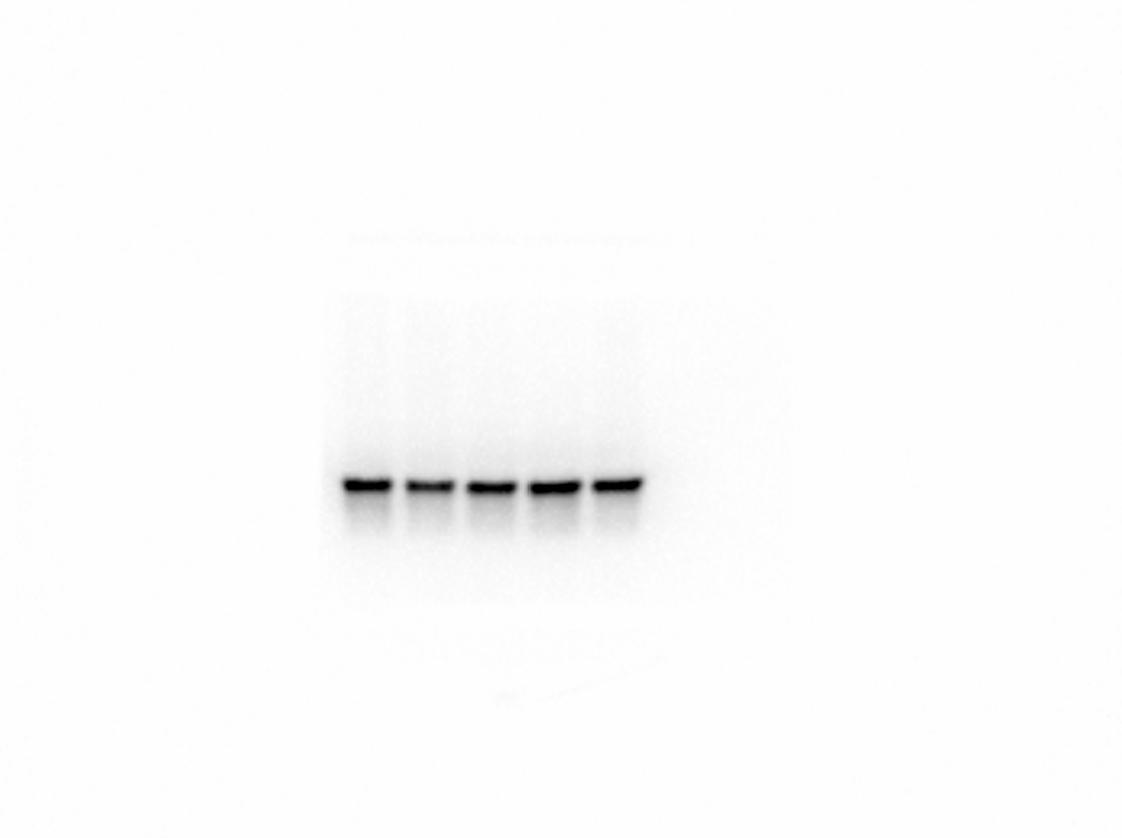

Supplement: Figure 1—figure supplement 1—source data 1. — Images are divided into two folders – one for each technical replicate. [file elife-66869-fig1-figsupp1-data1.zip › Set 2/Blot 2.4_PKAC.tif]

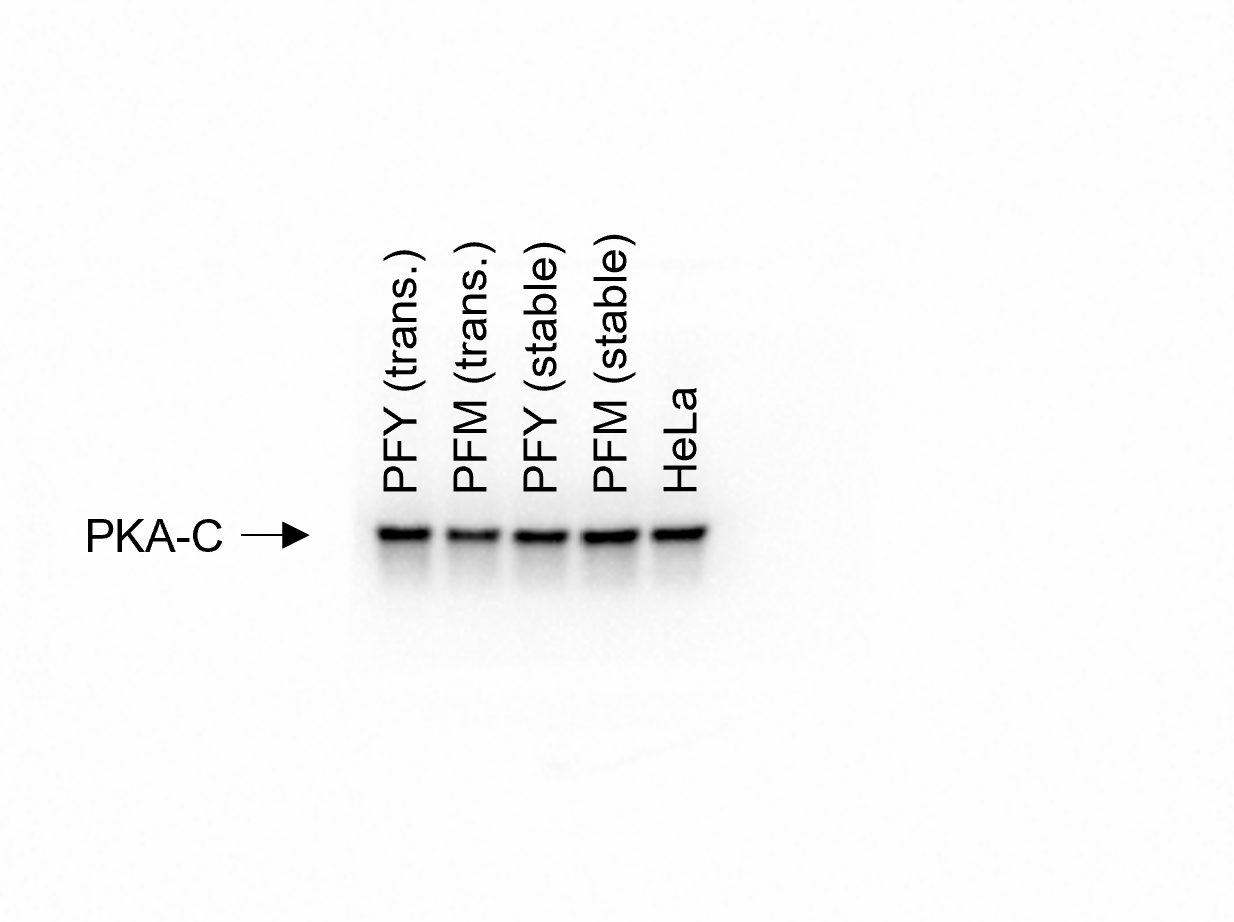

Supplement: Figure 1—figure supplement 1—source data 1. — Images are divided into two folders – one for each technical replicate. [file elife-66869-fig1-figsupp1-data1.zip › Set 2/Blot 2.4_PKAC_labeled.tif]
